# 真实世界维奈克拉联合去甲基化药物治疗较高危骨髓增生异常综合征疗效及安全性分析

**DOI:** 10.3760/cma.j.cn121090-20230926-00136

**Published:** 2024-02

**Authors:** 清妍 高, 冰 李, 士强 曲, 丽娟 潘, 蒙 焦, 金影 赵, 泽锋 徐, 志坚 肖, 铁军 秦

**Affiliations:** 1 中国医学科学院血液病医院（中国医学科学院血液学研究所），血液与健康全国重点实验室，国家血液系统疾病临床医学研究中心，细胞生态海河实验室，天津 300020 State Key Laboratory of Experimental Hematology, National Clinical Research Center for Blood Diseases, Haihe Laboratory of Cell Ecosystem, Institute of Hematology & Blood Diseases Hospital, Chinese Academy of Medical Sciences & Peking Union Medical College, Tianjin 300020, China; 2 天津医学健康研究院，天津 301600 Tianjin Institutes of Health Science，Tianjin 301600, China

**Keywords:** 维奈克拉, 去甲基化药物, 骨髓增生异常综合征, 真实世界, Venetoclax, Hypomethylating agents, Myelodysplastic syndromes, Real world

## Abstract

**目的:**

探讨维奈克拉（VEN）联合去甲基化药物（HMA）治疗较高危［修订后国际预后评分系统（IPSS-R）评分>3.5分］的骨髓增生异常综合征（MDS）的疗效及安全性。

**方法:**

纳入2021年3月至2022年12月于中国医学科学院血液病医院连续收治的共计45例应用VEN联合HMA方案治疗的较高危MDS患者，回顾性收集并分析临床资料，主要包括性别、年龄、MDS亚型、IPSS-R评分、治疗方案及疗效等，采用Kaplan-Meier法和Cox回归模型进行生存预后的单因素及多因素分析。

**结果:**

①共计45例MDS患者，其中91％患者为IPSS-R评分高危或极高危患者。按照国际工作组（IWG）2023版修订评价标准：总缓解率（ORR）为62.2％（28/45），完全缓解（CR）率为33.3％（15/45）。25例初治患者ORR为68％（17/25），CR率为32％（8/25）。20例非初治MDS患者ORR为55％（11/20），CR率为35％（7/20）。患者达最佳疗效中位周期数为1（1～4）个。②中位随访时间189 d，中位总生存（OS）期为499（95％ *CI* 287～711）d，患者死亡多因本病进展。VEN应答者中位OS期明显长于无应答者（499 d对228 d，*P*<0.001）。③多因素分析显示IPSS-R评分、对治疗反应为影响OS的独立预后因素；存在SETBP1基因突变可能延长患者住院时间（51.5 d对27 d，*P*＝0.017）。

**结论:**

VEN联合HMA治疗较高危MDS患者存在临床获益，但需警惕治疗过程中发生严重血细胞减低等不良反应。

## Abstract (en)

**Objective:**

To investigate the efficacy and safety of combining venetoclax （VEN） with hypomethylated drugs （HMA） in the treatment of higher-risk （IPSS-R score >3.5） myelodysplastic syndromes （MDS）.

**Methods:**

From March 2021 to December 2022, forty-five MDS patients with intermediate and high risk were treated with VEN in combination with HMAs. Clinical data were collected and analyzed retrospectively, including gender, age, MDS subtype, IPSS-R score, treatment regimen, and efficacy, etc. Kaplan-Meier method and Cox regression model were used to analyze univariate and multivariate of survival prognosis.

**Results:**

①Forty-five patients with MDS, including ninety-one percent were classified as high or very high risk. According to the 2023 consensus proposal for revised International Working Group response criteria for higher-risk MDS, the overall response rate （ORR） was 62.2％ （28/45）, with the complete response rate （CR） was 33.3％ （15/45）. For twenty-five naïve MDS, the ORR was 68％ （17/25） and the CR rate was 32％ （8/25）. In nonfirst-line patients, the ORR and CR were 55％ （11/20） and 35％ （7/20） respectively. The median cycle to best response was 1（1−4）. ②With a median followup of 189 days, the median overall survival（OS） time was 499 （95％ confidence interval, 287−711） days, and most patients died from disease progression. Responders had a significantly better median OS time than nonresponders （499 days *vs* 228 days, *P*<0.001）. Multifactor analysis revealed that IPSS-R score and response to treatment were independent prognostic factors for OS; the presence of SETBP1 gene mutations was associated with a longer hospital stay （51.5 days *vs* 27 days, *P*＝0.017）.

**Conclusion:**

There is clinical benefit of venetoclax in combination with hypomethylated agents in patients with higher-risk MDS, but adverse events such as severe hypocytopenia during treatment should be avoided.

骨髓增生异常综合征（MDS）为一组起源于造血干细胞的异质性髓系肿瘤[1]–[2]，其治疗选择根据国际预后评分系统（IPSS）、修订后的IPSS（IPSS-R）评分以及疾病的分子突变特征确定的疾病预后风险来制定[3]。目前，allo-HSCT为较高危组MDS（HR-MDS）唯一治愈手段。去甲基化药物（HMA）为不适合移植的HR-MDS患者标准治疗选择，但疗效有限[4]–[5]。

Ⅲ期临床试验数据显示阿扎胞苷（AZA）单药治疗高危MDS有效率为49％，中位OS期为24.5个月[4]，但后续临床试验及真实世界数据均未达其疗效，中位OS期波动于10.1～21.1个月[6]。Sekeres等[7]曾尝试来那度胺等药物联合HMA治疗高危MDS，疗效并不优于HMA单药治疗。维奈克拉（VEN）作为口服可吸收的小分子选择性BCL2蛋白抑制剂，联合AZA或地西他滨（DAC）治疗老年髓系白血病有效且不良反应可耐受[8]。高危MDS存在BCL2蛋白过度表达，促凋亡蛋白（BAX/BAD）与抗凋亡蛋白（BCL2/BCL-XL）比例显著降低，从而导致细胞凋亡抵抗[3],[9]–[10]。临床前研究进一步证实BCL2蛋白抑制剂对高危MDS患者骨髓干祖细胞具有特异性毒性[9]。基于上述，Wei等[11]及Zeidan等[12]尝试应用HMA联合VEN治疗高危MDS。本研究回顾性分析45例接受VEN联合HMA治疗的较高危MDS患者，探索其疗效及不良反应。

## 病例与方法

1. 病例：自2021年3月至2022年12月中国医学科学院血液病医院连续收治共120例髓系肿瘤患者接受了包含VEN方案的治疗，其中45例MDS患者接受VEN联合HMA治疗。本研究回顾性收集并分析45例MDS患者临床资料。MDS的诊断符参照WHO 2016诊断标准[13]。MDS预后分层基于IPSS-R评分系统[14]。复发/难治性MDS定义为接受HMAs≥4个周期后出现疾病复发或进展[15]。所有患者基线均进行二代测序基因突变检测及染色体核型分析，检测结果纳入既往文献报道与疾病密切相关基因突变及可能相关的基因突变进行分析。

2. 治疗方案：所有患者均接受了至少1个周期VEN联合HMA治疗，VEN每日400 mg，从治疗第1天起给予，持续给予14 d或更短的时间（根据药物不良反应及耐受性进行调整），VEN治疗剂量根据同期药物相互作用进行调整，其中20例患者（44.4％）因联合使用唑类抗真菌药物减量。去甲基化药物AZA剂量为50～75 mg·kg^−1^·d^−1^×7 d或DAC剂量为20 mg/m^2^×5 d。治疗以28 d为1个周期，直到无法耐受、疾病进展或死亡，允许因不良事件（AE）或血细胞计数恢复情况延迟用药。

3. 疗效评价及安全性分析：为与既往文献疗效可比较，及可用于未来疗效比较，本研究患者疗效参照2023年国际工作组（IWG）修订的高危MDS疗效评价标准[16]和2006修订版本[17]分别进行评价。2023版评价标准定义总体缓解率（ORR）包括完全缓解（CR）率，部分缓解（PR）率，完全缓解伴有限的血细胞恢复［CR_L_，包括单系完全缓解（CR_uni_）和两系完全缓解（CR_bi_）］率，完全缓解伴部分血液学反应（CR_h_）率和血液学改善［HI，包括红系血液学改善（HI-E），血小板血液学改善（HI-P）和中性粒细胞血液学改善（HI-N）］率。2006版标准包括CR、PR、骨髓完全缓解（mCR）、疾病稳定（SD）、治疗失败及复发，未定义ORR，且无CR_L_及CR_h_定义；参照既往文献[18]，定义ORR为CR率+mCR率+PR率。AE根据WHO推荐的CTCAE 5.0版进行评价[19]。

4. 随访：采用住院复查、门诊复查及电话随访，末次随访日期为2023年4月25日，中位随访时间为189（24～575）d。随访结局事件为死亡，总生存（OS）期定义为开始应用VEN时间至（因任何原因）死亡的时间。

5. 统计学处理：应用SPSS 25.0软件进行统计学分析。计数资料采用例数（百分比）表示，计量资料采用*M*（范围）表示，连续变量根据正态性与否分别运用*t*检验或非参数Mann-Whitney检验进行组间比较，采用卡方检验比较两组间缓解率，相关性分析对连续变量采用Pearson检验，多组变量比较采用Kruskal-Wallis单因素方差分析，采用Kaplan-Meier法和Cox回归模型进行生存预后的单因素及多因素分析。*P*<0.05为差异有统计学意义。

## 结果

1. 临床特征：如表1所示，45例MDS患者中位年龄为56（23～76）岁，34例（75.6％）患者美国东部肿瘤协作组（ECOG）体能状态评分为0～1分。91.0％（41/45）IPSS-R危险度分层为高危及极高危MDS，4例为IPSS-R中危组，评分≥4.5分；42例（93.3％）骨髓基线原始细胞比例≥5％。25例（55.6％）为初治MDS患者；20例（44.4％）为非初治患者，既往接受过HMA治疗，中位HMA治疗周期为5.5（1～15）个。20例非初治患者中13例可评为复发/难治性MDS（既往应用HMA周期≥4个）。5例（11.1％）患者既往有血液系统疾病。基线基因突变中位数为3（0～6）个，排名前13位基因依次为TP53（14/45，31.1％）、ASXL1（10/45，22.2％）、RUNX1（10/45，22.2％）、DNMT3A（7/45，15.6％）、U2AF1（6/45，13.3％）、DDX41（5/45，11.1％）、STAG2（5/45，11.1％）、SETBP1（4/45，8.9％）、NPM1（4/45，8.9％）、IHD2（3/45，6.7％）、SRSF2（3/45，6.7％）、PHF6（3/45，6.7％）、ARID2（3/45，6.7％）。22例（48.9％）患者染色体核型正常，IPSS-R预后中等核型8例（17.8％），13例（28.9％）患者为复杂染色体核型，2例（4.4％）为IPSS-R预后差核型。

**表1:** 45例接受维奈克拉联合去甲基化药物治疗骨髓增生异常综合征患者的临床特征［例数（％）］

临床特征	总体（45例）	初治（25例）	非初治（20例）	*P*值
年龄[岁，*M*（范围）]	56（23~76）	54（23~74）	58.5（39~76）	0.023
性别				0.254
男	20（44.4）	13（52.0）	7（35.0）	
女	25（55.6）	12（48.0）	13（65.0）	
ECOG体能评分				0.961
0分	13（28.9）	7（28.0）	6（30.0）	
1分	21（46.7）	12（48.0）	9（45.0）	
2分	9（20.0）	5（20.0）	4（20.0）	
3分	1（2.2）	0（0）	1（5.0）	
4分	1（2.2）	1（4.0）	0（0）	
基线骨髓原始细胞比例				0.390
<5%	3（6.6）	3（12.0）	0（0）	
EB1（5%~9%）	4（8.9）	2（8.0）	2(10.0)	
EB2（10%~19%或有Auer小体）	38（84.4）	20（80.0）	18(90.0)	
IPSS-R预后分层				0.403
中危	4（8.9）	3（12.0）	1（5.0）	
高危	17（37.8）	11（44.0）	6（30.0）	
极高危	24（53.5）	11（44.0）	13（65.0）	
存在前驱血液病史	5（11.1）	3（12.0）	2（10.0）	0.831
染色体核型				0.730
正常核型	22（48.9）	11（44.0）	11（55.0）	
复杂核型	13（28.9）	7（28.0）	6（30.0）	
IPSS-R预后中等核型	8（17.8）	6（24.0）	2（10.0）	
除复杂核型外IPSS-R预后差及极差核型	2（4.4）	1（4.0）	1（5.0）	

注 ECOG：美国东部肿瘤协作组；EB1：原始细胞增多1型；EB2：原始细胞增多2型；IPSS-R：修订后的国际预后评分系统

2. 疗效分析：如表2所示，截至末次随访，患者接受VEN联合HMA治疗的中位周期为2（1～10）个。按照2023 IWG评价标准：45例患者ORR为62.2％（28/45），CR率为33.3％（15/45），CR_L_率为13.3％（6/45），CR_h_率为2.2％（1/45），HI率为13.3％（6/45）。25例初治MDS患者ORR为68％（17/25），CR率为32％（8/25）；20例非初治MDS患者ORR为55％（11/20），CR率为35％（7/20）。患者达最佳疗效中位周期为1（1～4）个。

**表2:** 45例接受维奈克拉联合去甲基化药物治疗骨髓增生异常综合征患者疗效［例数（％）］

疗效评价标准	总体（45例）	初治（25例）	非初治（20例）	*P*值
2023版疗效评价标准				
总体反应	28（62.2）	17（68.0）	11（55.0）	0.437
CR	15（33.3）	8（32.0）	7（35.0）	
CR_L_	6（13.3）	5（20.0）	1（5.0）	
CR_uni_	2（4.4）	2（8.0）	0（0）	
CR_bi_	4（8.8）	3（12.0）	1（5.0）	
CR_h_	1（2.2）	0（0）	1（5.0）	
HI	6（13.3）	4（16.0）	2（10.0）	
2006版疗效评价标准				0.581
总体反应	24（53.3）	14（56.0）	10（50.0）	
CR	15（33.3）	8（32.0）	7（35.0）	
mCR	9（20.0）	6（24.0）	3（15.0）	
mCR伴HI	8（17.8）	5（20.0）	3（15.0）	
mCR不伴HI	1（2.2）	1（4.0）	0（0）	
PR	0（0）	0（0）	0（0）	
SD	8（17.8）	7（28.0）	1（5.0）	

注 CR：完全缓解；CR_L_：完全缓解伴有限的血细胞恢复；CR_uni_：完全缓解伴单系完全缓解；CR_bi_完全缓解伴两系完全缓解；CR_h_：完全缓解伴部分血液学反应；HI：血液学改善；mCR：骨髓完全缓解；PR：部分缓解；SD：疾病稳定

按照2006版疗效标准，45例患者ORR为53.3％（24/45），其中CR率为33.3％（15/45），mCR率为20％（9/45），mCR患者88.9％获得HI（8/9）。25例初治MDS患者ORR为56％（14/25），CR率为32％（8/25）；20例非初治MDS患者ORR为50％（10/20），CR率为35％（7/20）。

共12例患者接受allo-HSCT（10例亲缘/同胞半倍体，2例同胞全合），其中包括2例患者为VEN治疗失败，10例患者为对VEN治疗有反应。截至末次随访，2例死亡，1例为VEN治疗失败者并粒细胞原发性植入失败，死于重症感染；1例为获得HI反应者，移植半年后复发并死于肺感染。10例患者存活，3例发生急性GVHD，1例发生慢性GVHD。

中位随访时间189 d，中位OS期为499（95％ *CI* 287～711）d。死亡13例，10例患者因本病死亡，1例因脑出血死亡，2例因移植后并发症死亡。VEN应答者中位OS期为499（95％ *CI* 236～762）d，无应答者中位OS期为228（95％ *CI* 166～290）d，两者中位OS期差异有统计学意义（*P*<0.001）。初治MDS组中位OS期未达；非初治MDS组中位OS期为343（95％ *CI* 243～443）d。VEN治疗后行移植者中位OS期未达，但与未移植者中位OS期［343（95％ *CI* 253～433）d］比较差异无统计学意义（*P*＝0.285）（图1）。单因素分析显示IPSS-R评分、TP53突变、复杂核型及对治疗反应可影响患者OS，年龄、ASXL1、RUNX1、DNMT3A和DDX41等突变对OS无影响；多因素分析显示IPSS-R评分（*HR*＝3.68，95％ *CI* 1.455～9.316，*P*＝0.006）及对治疗的反应（*HR*＝99.720，95％ *CI* 4.881～2 037.212，*P*＝0.003）为影响OS的独立预后因素。

**图1:**
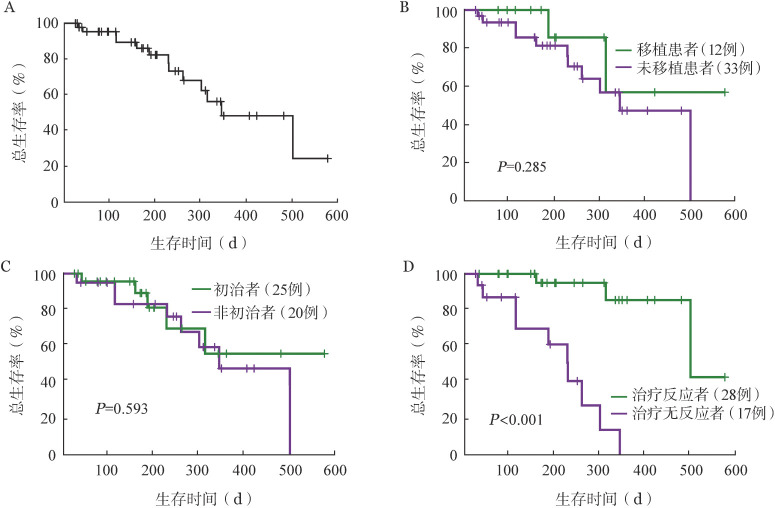
维奈克拉联合去甲基化药物治疗骨髓增生异常综合征患者生存分析 A 总生存曲线；B～D 移植、是否初治、治疗反应对生存的影响

TP53突变是本研究中患者最常见基因突变，57.1％（8/14）TP53突变患者获得疗效，包括3例CR、1例CR_bi_、1例CR_h_和3例HI，但2例患者短期（<3个月）复发并死亡。复杂核型患者ORR为53.8％（7/13），包括3例CR，1例CR_bi_和3例HI。

3. 安全性分析：共获得44例患者AE数据记录。最常见的3～4级AE为血细胞减少（图2），发生于93.2％（41/44）患者，第二常见AE为感染（37/44，84％），其他AE包括恶心（4/44，9.1％）、血清胆红素升高（4/44，9.1％）、丙氨酸转氨酶升高、天冬氨酸转氨酶升高（2/44，4.5％）、腹泻（1/44，2.2％）、颅内出血（1/44，2.2％）、皮疹（1/44，2.2％）、骨痛（1/44，2.2％）。8例患者在治疗过程中曾因AE中断VEN治疗，原因为严重感染和血细胞减少。1例患者因颅内出血AE死亡。

**图2:**
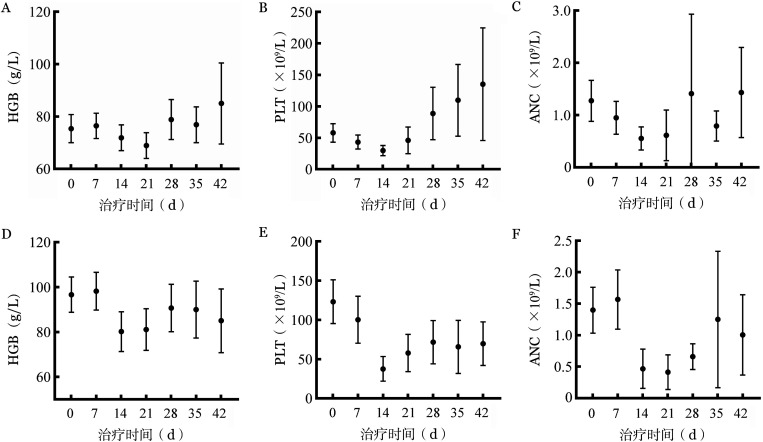
维奈克拉联合去甲基化药物治疗骨髓增生异常综合征过程中外周血细胞计数变化 A～C 第1疗程HGB、PLT和ANC变化［平均值（95％ *CI*）］；D～F 后续疗程HGB、PLT和ANC变化［平均值（95％ *CI*）］

4. 治疗剂量及间隔：45例患者中20例（44.4％）因联合应用唑类抗真菌药物，接受VEN每日剂量100 mg联合AZA治疗。15例（33.3％）患者接受VEN每日400 mg联合AZA治疗，10例（22.2％）患者接受VEN每日400 mg联合DAC治疗。血药浓度分析显示：VEN+AZA+唑类抗真菌药物组VEN谷浓度（中位2 376.5 ng/ml）高于其他两组（AZA+VEN 400 mg组，中位谷浓度983.2 ng/ml；DAC+VEN 400 mg组，中位谷浓度980.0 ng/ml；*P*＝0.001），而三组患者VEN血药浓度峰值差异无统计学意义。

本研究中，第1～2个周期间隔的中位时间为39（28～67）d，第2个周期后疗程间隔中位时间为41（28～97）d。因后续疗程受患者依从性等影响，本研究分析影响第1～2个周期间隔可能因素。结果显示基线血细胞计数、骨髓增生程度、纤维化程度、染色体核型、基因突变负荷及种类、VEN血药浓度均与治疗周期间隔时间无关。多因素分析显示SETBP1基因突变可延长住院时间（*P*＝0.017），存在SETBP1基因突变患者的中位住院时间为51.5（29～68）d，而无突变者为27（7～98）d。

## 讨论

本研究为目前我们所知国内应用VEN联合HMAs治疗MDS最大队列报道。研究中绝大多数（91％）患者为高危/极高危MDS，93.3％为原始细胞增多（EB）亚型，患者预后不良。我们采用2023年IWG高危MDS疗效评价标准修订版进行疗效评价。初治MDS患者ORR为68％，CR率为32％，CR_L_为20％，HI为16％；而非初治MDS患者ORR为55％，CR率为35％，CR_L_率为5％，CR_h_ 为5％，HI为10％。中位随访时间189 d，初治MDS组中位OS期未达；非初治MDS组中位OS期为343（95％ *CI* 243～443）d。无论初治及非初治MDS患者，VEN联合HMA治疗存在临床获益。

此前，VEN联合阿扎胞苷治疗初治较高危MDS的Ⅰb期临床试验数据显示：按照2006版疗效评价标准，57例患者ORR为77％，包括CR率为42％，mCR率为35％（其中40％ mCR患者获得HI）；中位OS期未达[20]。对于难治/复发MDS患者，CR率为7％，mCR率为32％，36％患者可脱离红细胞或血小板输注依赖，mCR患者中43％获得HI；中位OS期为12.6个月[15]。本研究按照2006版疗效评价标准，初治组ORR为56％（CR率为32％，mCR率为24％），mCR患者83.3％获得HI；非初治组ORR为50％（CR率为35％，mCR且HI率为15％）。本研究中初治患者疗效稍劣于Ⅰb期临床试验，仍优于既往文献报道HMAs单药疗效：ORR为30％～63.5％，CR为12％～21％[21]–[25]，中位OS期为10.1～21.1个月[6]。目前，另外2项Ⅰ～Ⅱ期临床试验公布的结果VEN联合HMA治疗高危MDS ORR为75％～87％（包含初治及难治复发），而真实世界中数据显示ORR为67.5％～87％，初治患者ORR为75％～77％，非初治ORR约62％[26]–[29]。因均为小样本研究，且结果多混杂初治及非初治患者，VEN联合HMA治疗较高危MDS真实世界疗效尚需更大样本数据。

本研究中，患者达最佳疗效中位周期数为1（1～4）个，与既往文献结果一致[15],[28]，与单药HMA相比[4]，联合治疗可使患者更早获得疗效。多因素分析显示IPSS-R评分及对治疗反应为影响OS的独立预后因素。Zeidan等[15]报道MDS常见突变如ASXL1、RUNX1和DNMT3A等对VEN联合HMA治疗患者生存无影响，而TP53突变者应答率较低、OS期较短。本研究中携带TP53突变及复杂染色体核型等高危遗传学异常患者有效率约50％。由于例数限制，VEN联合HMA治疗对高危遗传学异常MDS患者疗效尚需更大规模数据验证。值得注意的是，Gangat等[30]研究中70％患者应用28 d剂量VEN，结果显示ASXL1突变（突变者中位OS期未达，无突变者10.2个月）及获得CR疗效（CR者OS未达，无CR者10.2个月）对OS具有独立影响。

血细胞减少仍为本研究患者最常见AE（93％），相关文献报道HMA单药临床试验报道粒细胞减少发生率为89％，血小板减少发生率93％～96％，贫血发生率64％～77％[4]，联合治疗并未增加血细胞减少发生率。本研究中8例患者因AE中断VEN治疗，主要原因包括严重感染和血细胞减少。因血细胞减少为最常见AE，影响患者后续治疗，我们进一步探索影响患者治疗周期间隔的因素。基线因素如年龄、基线血细胞计数、骨髓增生程度、纤维化程度、染色体核型、基因突变负荷及种类等未影响治疗间隔。进一步分析影响患者住院时间的因素，结果显示SETBP1基因突变可延长患者住院时间。

综上所述，VEN联合HMA治疗我国较高危MDS总体耐受良好，ORR优于既往HMA单药，治疗后血细胞减少仍为患者最常见AE，需警惕并密切监测。IPSS-R评分及对治疗的反应为影响OS独立预后因素。但本研究样本数有限且为回顾性分析，随访时间尚短，未来仍需更大样本量的前瞻性随机对照临床试验来进一步验证VEN联合HMA在高危MDS的有效性和安全性。
